# Open visitation policy in intensive care units in Jordan: cross-sectional study of nurses' perceptions

**DOI:** 10.1186/s12912-022-01116-5

**Published:** 2022-12-02

**Authors:** Haya Ibrahim Ali Abu Maloh, Samiha Jarrah, Nabeel Al-Yateem, Fatma Refaat Ahmed, Mohannad Eid AbuRuz

**Affiliations:** 1 Faculty of Nursing, Applied Science Private University, Amman, Jordan; 2 Department of Nursing, College of Health Sciences, University of Sharjah, Sharjah, UAE; 3 Critical Care and Emergency Nursing Department, Faculty of Nursing, Alexandria University, Alexandria, Egypt

**Keywords:** Intensive care units, Nursing Management, ICU nurses, Family-centered care, Family nursing, Visitation policy

## Abstract

**Introduction:**

Intensive care unit patients and families experience significant stress. It creates frustrations, nervousness, irritability, social isolation for patients, anxiety, and depression for families. An open visitation policy with no time or duration limits may assist in reducing these negative experiences. However, most Jordanian and regional hospitals within the Middle-East and Northern Africa (MENA) have not implemented this strategy.

**Purpose:**

To evaluate nurse managers' and nurses' perspectives on the effects of an open visitation policy at intensive care units (ICUs) on patients, families, and nurses' care.

**Method:**

A cross-sectional, descriptive, and comparative survey design was used.

**Results:**

A total of 234 nurses participated in the study; 59.4% were males, and 40.6% were females. The mean of their age was 28.6 years, with a mean of 4.1 years of experience. Nurses generally had negative perceptions and attitudes toward the open visitation policy and its consequences on the patient, family, and nursing care.

**Conclusions:**

ICU managers and staff nurses did not favor implementing an open visitation in their units despite its known benefits, international recommendations, and relevance and compatibility with the local religious and cultural context. A serious discussion regarding this hesitation from the side of the healthcare professionals should be started to find a suitable solutions that consider the benefits of the open visitation policy and the challenges that prevent its implementation in the Jordanian and Arabic cultures.

## Introduction

Families of patients admitted to intensive care units (ICU) are exposed to several distressful events that are often experienced as anxiety, stress, and even depression [1–3]. Open visitation, is one of the strategies that managers and policymakers recommended to lessen this stressful experience. [1, 4, 5] Open visitation policy is imposing no restrictions on the time or duration of the visit, this policy's advantages and disadvantages differ from patient to family to ICU workers. [4, 6, 7] The main advantage of the open visiting policy is the positive psychological effect on the patient and the family because they are not separated for long periods, instead there is no restrictions on the duration of the visit. [4, 8–10] It helps patients by providing a support system, creating a more familiar environment and encouraging flexibility [8, 11]. The presence of family helps improve the patient's well-being and serves as a relief from hospital routine [12]. Open visitation is a significant element of composing a patient and family-centered approach and is considered a top priority by the American Association of Critical-Care Nurses [13, 14].

Some studies even suggested that the quality of life of survivors and relatives is positively influenced by a good relationship between them and the ICU team. Open visitation has been reported to strengthen and enhance this relationship [9, 15, 16] decrease anxiety and depression, and improve satisfaction among family members [7, 17]. Finally, among the reported benefits of open visiting to the healthcare professionals is that it increases their satisfaction as they provide more comprehensive care and the improved trust with the patient's relatives [9, 14].

Even though the attendance of family and friends may support patients during their stay in a very unfamiliar and stressful environment, the effect of open visiting hours on ICU staff is still controversial [6, 9, 18, 19]. The main disadvantage of open visiting from the perspective of healthcare professionals is the distraction that this approach can cause to care in the ICU and the increase in stress when handling patients in the presence of their family members for long hours per day [4]. Also, when being at the bedside, family members will need continuous information to be delivered, which may adversely affect the ICU care delivery [20].

The issue of open visitation is similarly relevant for Jordan and the surrounding region. In this region, the Islamic religion and Arab culture are dominant, with the family lying at the core of the social structure. Kinship, family structure, varying roles among family members, extended family, and support systems are distinctive features of the local culture and religion that must be incorporated into the competent, sensitive, and holistic nursing care [21]. Family members see visiting, being with, and caring for unwell relatives as a form of worship or a religious act. These are critical and sensitive subjects within the Islamic religion and Arabic culture. Therefore, family members may request to be present throughout the care of the critically ill or injured family to be with them, follow their religion, and ensure that their loved one receives the best possible care [22].

Open vistitation could directly impact nurses’ work environment and flow. Due to the serious conditions of the patients they care for, nurses are subject to considerable work-related stress and are more likely to feel compassion fatigue. Nurses are also burdened with a more workload. In these conditions, in addition to caring for critically ill patients, nurses must also tend to the numerous demands of visitors, which adds to compassion fatigue and lowers professional quality of life. ICU nurses have therefore being resistant to open visitation despite its advantages for patients and their families [4, 5, 7].

In conclusion, the dilemma is still present in the ICU setting as to which visiting policy adequately incorporates the family members into the plan of care of the patient while at the same time allowing the ICU workers to safely manage the care of the ICU patient without any conflict situation occurring [23]. To address this dilemma in the best possible manner, the beliefs and attitudes of ICU stakeholders such as managers, staff nurses, families, and patients need to be researched as their views will influence the type of visiting policy that will be implemented.

However, observations from inside the healthcare system in Jordan and the region indicated that this area is still unclear, unconsidered, and lacks a clear understanding of the stakeholders' views and care policies and guidelines. Therefore, this study will take an initial step and explore managers' and nurses' perspectives on the open visitation policy at ICUs in Jordan and its effects on patients, families, and their care.

### Research aim

To explore the perceptions of the mangers’ and nurses’ about the effect of the open visitation policy on patient care, family satisfaction, and nurses' abilities to provide care in the ICUs in Jordan.

## Methods

### Research design

A descriptive and comparative design was used to meet the objective of this study.

### Setting and sample

Five hospitals (one public hospitals and four private hospitals) that reflect the largest number of nurses working in the Critical Care Units in Jordan were selected to recruit the study participants. The selected hospitals represent the governmental, private, and academic sectors, the three main sectors that provide health services in the country. Nursemanagers and staff nurses in the Critical Care Units from the accessed hospitals such as Intensive Care, Coronary Care, Emergency, and Burn Units and working in the ICUs for more than one year were included in the study. The required sample size was calculated using G* Power analysis based on a medium effect size of 0.15, an α of 0.05, a power of 0.8 and independent t-test as a statistical test for analysis. Based on these assumptions, it was found that the needed sample size was 230. Therefore, at the conclusion of the study, 234 nurses were recruited conveniently.

### Data collection

Data were collected from June 2021 to October 2021 using structured, self-administered questionnaires. A list of all nurse mangers and staff nurses in the selected settings was prepared and recruitment was managed via appointments arranged by the researcher and trained data collector. The researcher and trained data collector informed the respondents about the nature, and purpose of the study and that participation was voluntary. They were further told that they had the right to withdraw at any time they wished. The researcher collected data from public and private hospitals with the help of a data collector who has no conflict of interest with the participants and was trained by the researcher on the data collection procedure to ensure consistency. In addition, the researcher and research assistant were available to participants to clarify any inquiries. The questionnaires required 10–15 min to be completed.

### Instrument

First, the current visitation practices at ICU questionnaire was used. This questionnaire was developed by the researchers and included 7 questions about the present visitation at the ICU in participants' facilities. Questions in the questionnaire covered four domains: (1) restrictions on visits which includes four question; visiting duration allowed currently, number of visitors allowed at one time, limit on who visits, the maximum visiting permitted time per visitor. (2) Exceptions in the visiting policy to be made, (3) specific times during the day when no visitors are allowed, and (4) availability of a written visiting policy for the ICU.

Second, the Beliefs and Attitudes toward Visitation in ICU Questionnaire (BAVIQ) was used [17, 19]. The questionnaire assesses ICU nurses' beliefs and attitudes toward different aspects of visitation and visiting hours. The BAVIQ questionnaire consists of two parts and is divided into four subscales; the first part is about nurses' beliefs about the consequences of visitation on the patient (Qs 1 – 9), family (Q 10 and 11), and nursing care. The second part is nurses' attitudes towards visiting, and these included questions twenty-one to thirty-two. The questionnaire uses a five-point Likert scale with answer options ranging from strongly disagree to strongly agree. The validity and reliability of the questionnaire have been confirmed in previous studies [24, 25], which reported a Cronbach's alpha coefficient of around 0.78.

### Ethical consideration

Institutional Review Board (IRB) approval was obtained from Institutional Review Board (IRB), Faculty of Nursing, Applied Science Private University; (IRB No. Student 1–5- 2021–1). Permission from the corresponding author was taken to use the BAVIQ. It was clarified to the nurses that participation is voluntary and that returning a filled questionnaire means the approval of the nurses to participate in the study. The study was anonymous; no identifiers were required from the participants.

### Data analysis

The Statistical Package for the Social Sciences (SPSS 25) was used to analyze the data. Descriptive statistics were used to describe the characteristics of the study sample. Descriptive statistics of the variables (i.e., frequency, percentage, mean, and standard deviation) were reported for the current visitation policy at ICU. Nurses' beliefs about the consequences of visitation on the patient, family, and organization of care were reported as well as their attitudes towards visiting. An independent samples t-test was conducted to compare nurses' attitudes toward visiting and the consequences of visitation on the patient, family, and nursing care for private and public hospitals. The significance level (P-value) of less than 0.05 was considered a statistically significant result.

## Results

### Participants' characteristics

A total of 234 nurses participated in the study. Among participants, 139 were males, and 95 were females. The mean age of participants was 28.6 ± 4.4 years. More than half of the sample, 134 (57.3%), were from private hospitals, and almost all of them (99.1%) had a bachelor's degree in nursing, with a majority (88.0%) working as clinical bedside nurses. Finally, participants have a mean experience duration of 4.1 years, as shown in Table 1.

**Table 1:** Sociodemographic characteristics of the sample (*N* = 234)

	**Participant's characteristics**	**M ± SD or N (%)**
Age		28.6** ± **4.4
Gender	Male Female	139 (59.4) 95 (40.6)
Employment	Private hospital Public hospital	134 (57.3) 100 (42.7)
Education	Bachelor's degree Master's degree	232 (99.1) 2 (0.9)
Management position	No Management positions In-charge Nurse Head Nurse	206 (88.0) 25 (10.7) 3 (1.3)
Years of experience		4.1** ± **3.4

### Participants' responses regarding visiting policy

Findings related to the visitation policy are shown in Table 2; 35.9% of participants reported that visitation is currently limited to two visiting times per day, 38.9% of the total participants said that visitation is presently limited to two visitors at a time, and 50.0% responded that maximum visiting time currently is between 5–10 min. A 46.6% responded that exceptions in the visiting policy could be made when the patient is dying. While 55.1% responded that there were not any specific times during the day when no visitors were allowed. 57.7% of the participants respond that they do not have an official written visiting policy at their ICU.

**Table 2:** Participants' responses regarding visitation policy (*N* = 234)

	**Responses**	**N**	**(%)**
Number of visits /24 h	One Visiting time Two Visiting times Three Visiting times Four or more visiting times No Visiting time	79 84 33 13 25	(33.8) (35.9) (14.1) (5.6) (10.7)
Number of visitors /one time	One Visitor Two Visitors Three Visitors Four or more visitors No limit on visitors	64 91 16 8 55	(27.4) (38.9) (6.8) (3.4) (23.5)
Maximum visiting time	Less than 5 min 5–10 min 10–15 min 15–30 min No time limit	4 117 39 16 58	(1.7) (50.0) (16.7) (6.8) (24.8)
Exceptions to be made	Patient is dying Family has practical problems in complying with the policy Patient has emotional needs Healthcare workers have practical needs	109 51 44 30	(46.6) (21.8) (18.8) (12.8)
Specific times no visitors are allowed	No specific time During CPR During endorsement During Doctor Round During procedure During nursing care	129 54 19 13 12 7	(55.1) (23.1) (8.1) (5.6) (5.1) (3.0)
Availability of written policy at ICU	Yes No	99 135	(42.3) (57.7)

### Consequences of visitation on the patient, family, and nursing care

The item that received the highest score was "An open visiting policy interferes with direct nursing care." The five highest-rated consequences of the open visiting policy were about the undesired consequences of the policy on direct nursing care. Participants believed that this policy would interfere with the nursing care (Mean = 3.8), nurses would spend more time providing information to the family (Mean = 3.8), open visiting would interfere with the communication between the nurses (Mean = 3.7), it would increase the risk of errors (Mean = 3.7), and finally disturbs patients rest (Mean = 3.6). The first desired consequence of the policy was mentioned in the sixth rank. It was that the visitors can help the patient interpret information (Mean = 3.6) and that the visitation has a beneficial effect on the patient (Mean = 3.5). Then after that immediately, the negative consequences were reported again and included: "An open visiting policy makes nurses nervous because they are afraid to make a mistake" (Mean = 3.5), "the open visiting policy interferes with the adequate planning of the nursing care process" (Mean = 3.5), "the open visiting policy will create adverse hemodynamic responses in patients" (Mean = 3.4) and violate upon their privacy" (Mean = 3.4). The least rated item was that the open visiting policy is essential for the patient's recovery (Mean = 2.8). Table 3 presents all the items and their rating ordered from the top to the lowest.

**Table 3:** Means and standard deviations of the consequences of visitation on the patient, family, and nursing care (*N* = 234)

	**Statement**	**Mean**	**SD**
**1**	An open visiting policy interferes with direct nursing care	3.8	1.2
**2**	An open visiting policy makes nurses to spend more time in providing information to the family	3.8	1
**3**	An open visiting policy interferes with communication between nurses	3.7	1.1
**4**	An open visiting policy increases the risk of errors	3.7	1.1
**5**	Visitation disturbs the patient rest	3.6	0.9
**6**	Visitors can help the patient interpret information	3.6	1.1
**7**	Visitation has a beneficial effect on the patient	3.5	1
**8**	An open visiting policy makes nurses nervous, because they are afraid to make a mistake	3.5	1.2
**9**	An open visiting policy interferes with the adequate planning of the nursing care process	3.5	1
**10**	Visitation creates adverse hemodynamic responses in patients	3.4	1.1
**11**	An open visiting policy violate upon patient's privacy	3.4	1.2
**12**	An open visiting policy decreases family's anxiety	3.3	1.1
**13**	Visitation cause physiological stress for the patient	3.2	1
**14**	Visitation causes psychological stress for the patient	3.2	1.2
**15**	An open visiting policy exhausts family, because they feel forced to be with the patient	3.2	1.1
**16**	An open visiting policy contributes to the improvement of patient-centered care	3.2	1.1
**17**	Visitation is a helpful support for the care givers	3	1.2
**18**	An open visiting policy offers more comfort to the patient	2.9	1.2
**19**	An open visiting policy makes nurses feel controlled	2.9	1.3
**20**	An open visiting policy is important for the recovery of the patient	2.8	1.3

### Participants' responses regarding nurses' attitudes toward visiting

Participants generally had negative attitudes toward the open visiting policy. The highest-rated statement was that the available visiting policy must be adopted only when the patient is dying (Mean = 3.5) or strictly when a patient has emotional needs (Mean = 3.2). Also, participants highly rated the statement that a strict visiting policy must be adopted when a family has problems adhering to the policy (M = 3.1). Finally, and probably the most negative statement moderately rated by participants is that the open visiting policy should be adapted based on the culture or ethnicity of the patient (Mean = 2.8). The least rated statements by participants were that the number of visitors in a day, the number of visitors together, and the length of visit should not be limited (Mean ± SD = 2.2 ± 1.2, 2.3 ± 1.1, 2.3 ± 1.1 repectively) as shown in Table 4.

**Table 4:** Means and standard deviation of nurses' attitudes towards visiting (*N* = 234)

Attitudes towards visiting	Mean	SD
The visiting policy must be adapted when the patient is dying	3.5	1.2
Strict visiting hours must be adapted when the patient has emotional needs	3.2	1.2
When the patient is capable, he/she should have control in when, how long and how many visitors he/she can have	3.2	1.2
Strict visiting hours must be adapted when the family has practical problems adhering to the policy	3.1	1.2
The visiting policy must be adapted according to culture or ethnicity of the individual patient	2.8	1.2
An open visiting policy should be carried out in our unit	2.7	1.2
A strict starting hour is important, but the length of a visit can be flexible	2.7	1.1
The visiting policy must be flexible during the first 24 h of hospitalization	2.7	1.2
Everyone is allowed to visit, if it is approved by the patient	2.6	1.3
The length of a visit should not be limited	2.3	1.1
The number of people who are visiting the patient at the same time should not be limited	2.3	1.1
The number of visitors in a day (24 h) should not be limited	2.2	1.2

### Inferential analysis

An independent samples t-test showed that nurses in the different hospital sectors hold the same perceptions and attitudes toward the open visiting policy and its consequences on patients and nursing care (M [SD]; Patients:29.8 [4.3] vs. 29.4 [4.7], t = 0.75, *p* = 0.46; Nursing care: 30.5 [5.0] vs. 31.5 [4.9], t = 1.48, *p* = 0.14). However, these participants differ in their scores for the visitation effect on the family, and nurses working in a private hospital have the most positive perceptions and attitudes (M [SD]; 6.6 [1.4] vs. 6.0 [2.2], t = 2.583, *p* = 0.011).

## Discussion

When patients get treatment, especially in ICUs, healthcare institutions usually exclude family members or at least limit their presence or contribution to their patient's care; this is a finding of this study and has also been reported in previous studies. [22] Even during critical care and life-threatening events, family presence may be limited, and relatives may only be able to attend to their patient after treatment or when they are dying. As a result, patients and their family members are deprived of the opportunity to confront obstacles together, strengthen, empower, and soothe one another. [22] This study, therefore, would address a significant issue for the Jordanian and regional contexts. It has revealed the severe practice gap in the ICUs in Jordanian hospitals and the local culture and needs.

In Jordan and the surrounding Arab countries, Islamic beliefs and Arabic culture significantly impact all aspects of daily life and the population's needs. Moreover, these countries' social, economic, and healthcare systems also operate within the same context. [26] Therefore, comprehensive healthcare requires consideration of all essential patient and family aspects and needs, including religious and cultural considerations. [27] This is especially essential during difficult times such as illness, when Muslims usually rely more on religion. [20, 28, 29]

In Islam and among Muslims, life is regarded as a divine trust, and both health and illness are viewed as periods of stress requiring patience and resilience. Family members consider visiting, being with, and attending to the needs of ill individuals as a sort of worship or religious act. [29] In Islamic theology and Arabic culture, these are significant and delicate topics. Therefore, family members may request to be present during the care of critically ill or injured family members to be with them, stick to their religion, and guarantee that their beloved family member receives the best care possible. Understanding these demands and desires is crucial for providing appropriate, high-quality, and comprehensive care. Awareness of these culturally and religiously sensitive elements could help enhance cooperation and communication between healthcare providers and patients/family members. [30]

From a theoretical nursing point of view, Leininger (1996), a renowned nursing theorist and scholar, created a comprehensive care model to assist nurses in taking into account all of the factors that may affect care [31]. The model incorporated technology, religious and philosophical elements, kinship and social variables, cultural values, beliefs, ways of life, political and legal factors, economic issues, and educational aspects. Even in high-acuity, high-stress healthcare settings, such as critical care units, it is essential to provide complete, and competent treatment.

International evidence supports these views; studies have confirmed that the patient's family must be close by in times of health emergencies or injuries. Very early evidence that explored this issue reported that the most pressing requirements for families and relatives during their patient's stay at the hospital and critical illnesses were to have frequent contact with the patient, a sense of hope, and the belief that hospital staff cared about the patient, knowledge of the prognosis, and information, support, and reassurance from hospital staff, and to provide care and assistance [32, 33]. Despite the knowledge of the desire of family members to be close to ailing loved ones, especially during severe illnesses, and the early evidence in this regard, it appears that healthcare professionals in the Jordanian and similar Arabic contexts are still reluctant to adopt the family-centered approach and instead prefer to exclude family members during such times. Interestingly, this disparity exists even though healthcare professionals and patients share the same cultural and religious beliefs in many Arabic nations. However, in their practice, the wishes of family members to be present during the care provided for their loved ones in critical conditions are not consistently honored. In addition, there is a lack of regulations, guidelines, and research on the subject, even though the patient populations in these nations are primarily traditional and religious and have strong ties within families and extended social networks. [21, 34]

This reluctance of healthcare professionals to adopt a family-centered approach may be attributed to the context of intensive care units, which are highly complex and demanding. The management of patients requiring critical care typically relies on advanced technologies as life-sustaining treatment tools, which poses additional hurdles for healthcare personnel who must manage these technologies in the presence of family members if such a policy is adopted. In addition, the presence of family members in critical care settings and during treatment for critical illnesses is connected with several psychological and social issues and trauma, including shock, denial, guilt, and fear of losing the patient. In addition to providing direct patient care, healthcare professionals may confront several added duties, including providing psychological and emotional support for family members and taking their needs into account throughout patient care. [35, 36] For instance, if a family member cannot handle the stress of the circumstance, they may become another patient. Additionally, critical care practitioners must also prioritize the immediate requirements of their patients. [37] This may place the patient's family members in a peripheral position and give them the false sense that they are being ignored or are at risk if left unattended.

## Conclusions and recommendations

A holistic approach to care necessitates that health professionals acknowledge the significance of satisfying patients' and their families' needs. In the context of critical care, despite the challenges and added duties, clear communication and healthy relationships between healthcare personnel and family members are crucial. During illness, family members frequently fill a void that cannot be satisfied by medical professionals. Therefore, it is essential for personnel in critical care to establish collaborative connections with patients' family members, including exchanging information and offering required assistance.

In addition, certain religious beliefs and cultural values in Islam and Arabic cultures may provide support in the setting of severe illness, which may be helpful for healthcare practitioners. These religious beliefs and cultural values may also be linked to specific requirements (e.g., the need to continuously be with a patient to offer support and assistance and enable them to better comprehend and recover from a crisis). Ignoring such needs may compromise the quality of care and result in unintended effects. In contrast, family inclusion and open and regular communication with a patient's family, including discussing religious and cultural factors (as appropriate), can help identify and address special support needs. Clarification of these concerns and communication-specific training may consequently reduce the workload of critical care professionals and enhance the quality of care provided.

The literature has extensively explored the importance of familial and social elements in a family's presence during care for life-threatening diseases or injuries. Family members desire to assist and advocate for patients in critical times. In addition, maintaining family ties amid critical situations may positively affect the mental health of patients and their relatives. In the Arabic and Muslim environment, social bonds and family life are of utmost importance; all family members (both immediate and extended), friends and coworkers are expected to assist criticall ill family members. ICU visitation during COVID-19 was further compromised, it was possible again in May 2021 in our study settings, but with some restrictions (starting with a maximum of two family members for 1 h per day, and later on two persons for 2 h in the morning and two persons for 2 h in the evening). Critical care workers must recognize this responsibility and collaborate with each patient's family to ensure that this requirement can be provided while adhering to the environment's necessary limits.

### Limitations

Certain limitations of the current study merit mention. First, the present study settings and participants were selected conveniently from one geographical area. Even though this area contains most of the health services providers in the country, this issue may still affect the generalizability of the current findings. Second, the perceptions of nurses were only included. Future studies may explore the perceptions of families also.

## Data Availability

The datasets used and/or analyzed during the current study are available from the corresponding author on reasonable request.
